# Bovine xenograft pericardial patch use for definitive single stage repair of a large esophageal defect: a case report

**DOI:** 10.1186/s13019-021-01670-0

**Published:** 2021-10-13

**Authors:** Hani Shennib, Michelle Baribault, Richard Heuser

**Affiliations:** Department of Surgery, College of Medicine, University of Arizona, 6122 33rd St, Paradise Valley, Phoenix, AZ 85253 USA

**Keywords:** Esophageal perforation, Pericardial patch, Bovine pericardium, Case report

## Abstract

**Background:**

Large esophageal perforations are challenging and often treated with exclusion or resection. This case demonstrates the feasibility of definitive surgical repair of a large esophageal perforation using large bovine pericardial patch.

**Case:**

A patient with missed Boerhaave Syndrome underwent transesophageal echocardiography causing worsening perforation and sepsis. At thoracotomy and faced with a large esophageal defect, a large Bovine pericardial patch was used for repair with omentopexy. The patient recovered promptly and at 8 months was asymptomatic with satisfactory studies.

**Conclusion:**

Xenograft pericardium is available and widely used for vascular reconstructions. It’s use for primary repair of large esophageal perforations should be considered.

## Introduction

Non-malignant esophageal perforations are often iatrogenic or due to Boerhaave syndrome. Different nonoperative and operative treatments have been proposed over the years. Large perforations are often non-amenable to stenting and may require esophageal diversion or resection surgery. In moribund septic patients and particularly when delayed, such surgery carries a high morbidity and mortality [1–6]. Local thoracic flaps may not be good sealants, or of adequate sizes for repair of large esophageal defects. Vascular surgeons are accustomed to use of pericardial xenograft patches for vascular repair due to their availability, resistance to infection and durability, particularly when faced with large vascular defects. This case illustrates the feasibility of using a large bovine pericardial patch for definitive single stage repair of a large esophageal defect in a patient with delayed diagnosis of perforated esophagus from Boerhaave syndrome complicated by transesophageal echocardiography instrumentation.

## Case presentation

A 47-year-old Caucasian male with a past medical history of schizophrenia, alcohol and drug abuse presented to an outside facility with symptoms of recurrent emesis and epigastric pain. Abdominal CT scan and chest X-ray in the emergency room revealed right pneumothorax and a chest tube was placed. Soon after, the patients went into septic shock and required vasopressors, IV antibiotics and mechanical ventilation.

**Fig. 1:**
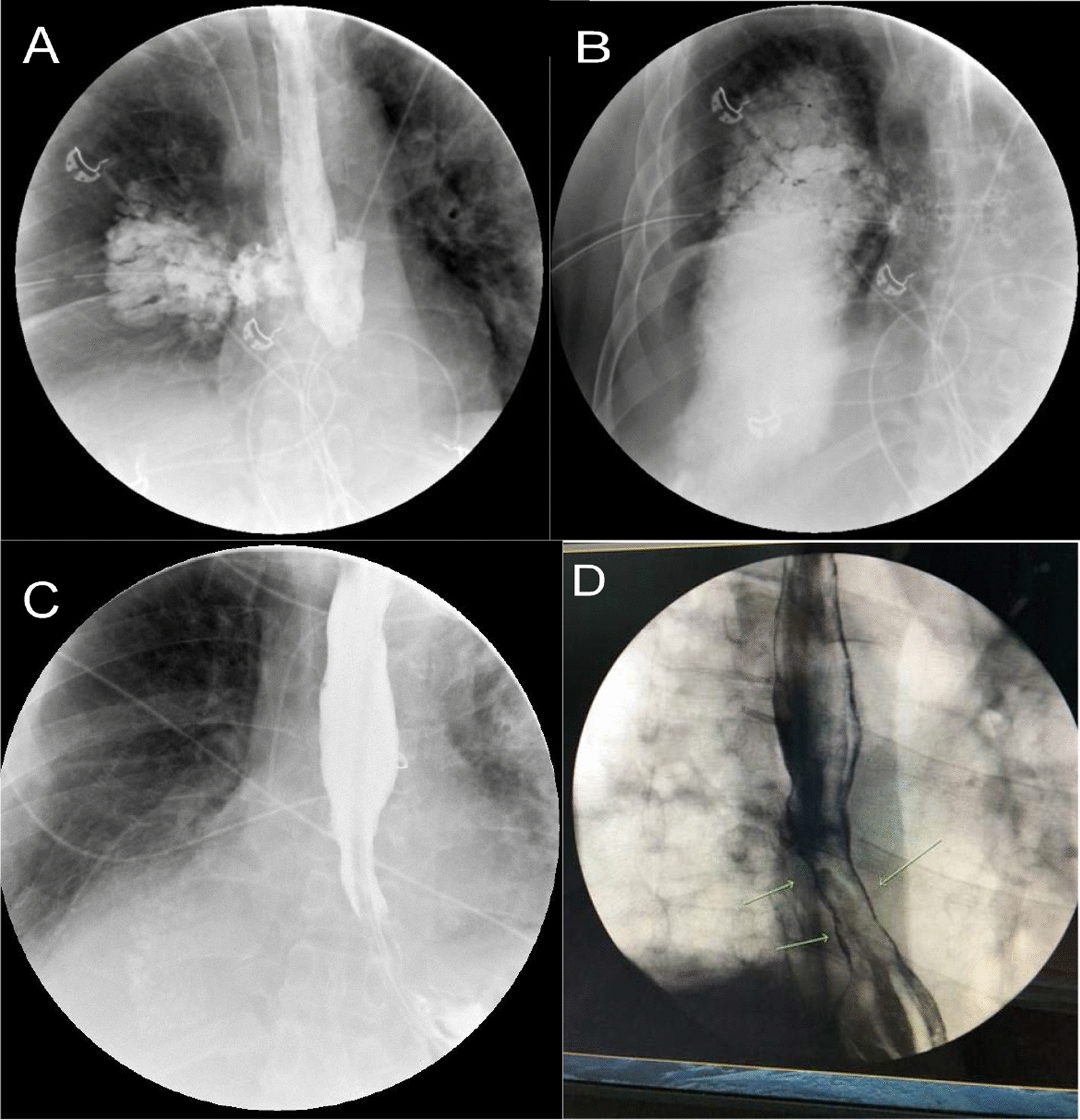
Gastrografin esophagram showing extravasation of contrast from lower esophageal perforation into right thorax (**A**, **B**). Follow up esophagram on post operative day 24 showing no leak (**C**). Follow up barium esophagram at 8 months showing non-obstructed flow into stomach (**D**)

**Fig. 2:**
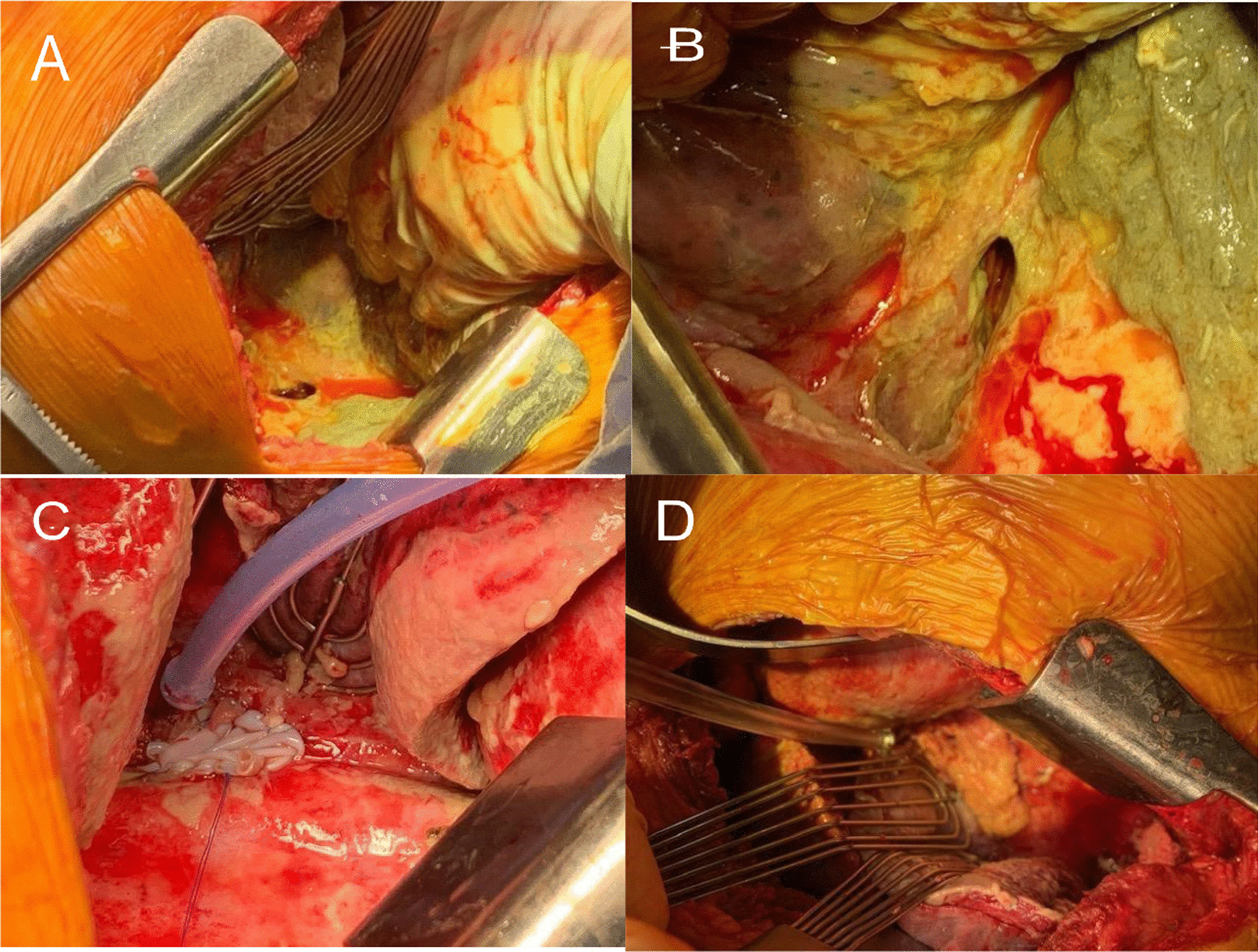
Initial defect measuring 5 × 3 cm (**A**). Measurement after debridement of necrotic edges 9 × 4 cm (**B**). Bovine pericardial patch covered defect (**C**). Pericardial patch covering the defect is reinforced with a tongue of omentum brought through the esophageal hiatus (**D**)

Esophagogastroscopy was deferred because of his instability. His blood cultures were positive for *staphylococcus hominis*. The following day a transesophageal echocardiogram to rule out valve endocarditis was negative. The patient appeared to improve on antibiotics but two days later his condition worsened again, and he was transferred to our facility. Upon arrival it was noted that there was copious thick mucinous output from the chest tube. A gastrografin study of the esophagus confirmed a sizable perforation at the lower esophagus (fig. 1A, B). Because of the appearance of a large esophageal defect with extensive pleural contamination, we did not consider stenting [5–7]. The patient was stabilized and taken to the operating room. A right posterolateral thoracotomy was done through the seventh intercostal space. The pleural space contained copious amounts of purulent fluid and gastric content which exuded from an exceptionally large esophageal defect in the lower third of the esophagus. The defect initially appeared to measure 5 × 3 cm in diameter, however after debridement of its necrotic edges it measured 9 × 4 cm (fig. 2A, B). Faced with the large defect, sepsis and delayed treatment, we entertained the idea of performing an esophageal exclusion surgery, however because of concern with his behavioral difficulties and the potential lack of compliance, we decided that a one stage attempt for definitive esophageal repair would be best for him. Because of his compromised status, we felt that he would not tolerate an esophagectomy and that an attempt to patch the large defect was justifiable. After careful debridement of the edges of the defect a bovine pericardial vascular patch (Edwards Lifesciences, model 4700, Irvine, Ca.) was tailored to the defect with an extra 2 cm around its edges and sewn using a running 3/0 prolene suture to the esophageal edges and surrounding mediastinal tissue for extra support. To further protect the pericardial patch repair, the greater omentum was mobilized using a separate upper midline abdominal incision, pulled through the esophageal hiatus and sewn using interrupted 3/0 Vicryl sutures to the tissues surrounding the pericardial patch repair (fig. 2C, D).

Gastrostomy and distal jejunostomy tubes were placed, and 2 nasogastric tubes were positioned proximal and distal to the esophageal repair. Two, size 32 chest tubes, were placed. Five days following his surgery and because of a worsening oxygen requirement, a chest CT was performed that showed an increasing right pleural effusion for which the patient underwent a redo thoracotomy and lung decortication.

**Fig. 3:**
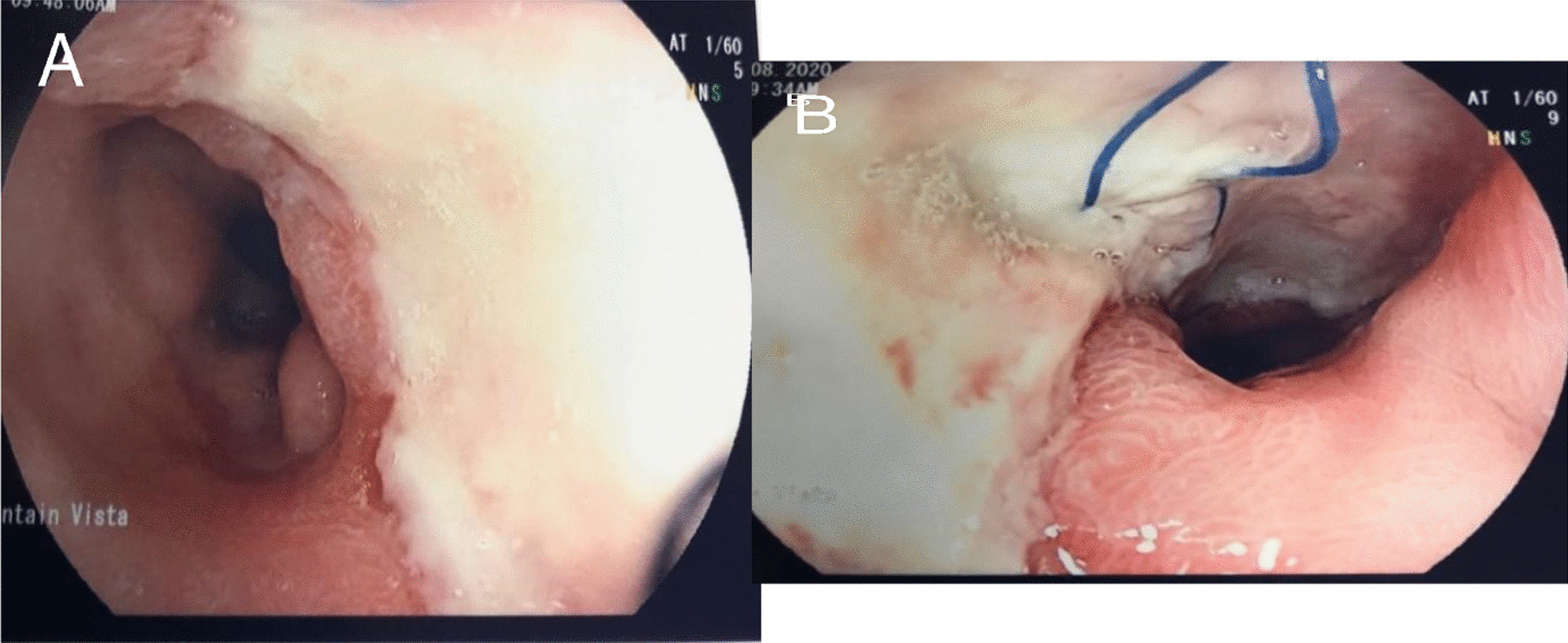
Endoscopic view of the pericardial patch repair 8 month postoperatively (**A**, **B**)

The patient was removed from the ventilator 3 days after and continued to improve thereafter. An esophageal gastrografin study performed on day 24 showed no leak (fig. 1C). His nasogastric tubes were removed, and he was started on clear liquids. He was discharged to a rehabilitation facility 28 days postoperatively. At 6 weeks follow up he was fully recovered and was eating a modified esophagectomy diet. His gastrostomy and jejunostomy tubes were removed. At 8 months follow up he was eating a normal diet with no difficulty and no complications with an esophageal barium study showing rapid non-obstructed flow of diet to the stomach and an endoscopic view of the pericardial patch (figs. 1D and 3A, B).

## Discussion

Bovine pericardium has been proven to be a durable, watertight and infection resistant material for use in penetrating infections of aortic and other vascular infections. It provides a generous readily available graft for patching serious and large vascular defects [8]. Most non-malignant esophageal perforations are iatrogenic or due to Boerhaave syndrome and most occur in the lower third of the esophagus [1–5]. Large and delayed esophageal perforations are particularly problematic with poor seal using stents and require resection with or without esophageal restoration of continuity which is an extensive operation in moribund patients. Exclusion procedures have high morbidity and prolonged tumultuous courses [1–5]. An increasing perforation length, delayed surgery, a higher Perforation Severity Score and a higher American Society of Anesthesiologists (ASA) score all lead to increased mortality in multivariate analysis [2, 3].

This case illustrates the feasibility of rapid and efficient repair of a large esophageal defect secondary to Boerhaave syndrome and complicated by transesophageal echocardiography manipulation resulting in septic shock and delayed treatment. There are occasional reports of the use of autologous pericardial tissue from the same patients to treat small esophageal perforations, however because of the large size of the defect observed in our patient, the option of using the limited size of the patient’s own pericardial tissue was not considered [9].

The ready availability of large patches of bovine pericardium in most thoracic operating rooms allows for prompt and simple repair in a short time with good seal following careful debridement of the esophageal defect edges. The resistance of biological tissue to infection and the characteristic compliance of the pericardium permits facile suturing with immediate watertight sealing. We also observed facile non-obstructed propagation of a swallowed bolus at the repair site due to this compliance. Reinforcement of the patch with omentopexy in this case provides further ability to control the periesophageal infection.

## Conclusion

The ability to perform definitive esophageal repair in moribund patients in one stage using bovine pericardium is undoubtedly an advantage and warrants further longitudinal studies to verify this technique’s effectiveness and durability.

## Data Availability

All data and information generated or analyzed during this study are included in this report.

## Abbreviations

CT: Computed tomography
IV: Intravenous
cm: Centimeters
